# Anticarcinogenic Effects of Gold Nanoparticles and Metformin Against MCF-7 and A549 Cells

**DOI:** 10.1007/s12011-024-04090-y

**Published:** 2024-02-15

**Authors:** Ali Yeşildağ, Halime Topal Kızıloğlu, Ebubekir Dirican, Elif Erbaş, Volkan Gelen, Adem Kara

**Affiliations:** 1 Department of Bioengineering, Faculty of Engineering and Architecture, Kafkas University, Kars, Turkey; 2 Department of Molecular Biology and Genetic, Faculty of Science, Erzurum Technical University, Erzurum, Turkey; 3 Department of Medical Biology, Faculty of Medicine, Bilecik Şeyh Edabali University, Bilecik, Turkey; 4 Department of Histology and Embryology Faculty of Veterinary Medicine, Atatürk University, Erzurum, Turkey; 5 Department of Physiology, Faculty of Veterinary Medicine, Kafkas University, Kars, Turkey

**Keywords:** A549, MCF7, Metformin, Ifosfamide, Gold Nanoparticles, Cell Cytotoxicity

## Abstract

Metformin is commonly prescribed to people with diabetes. Metformin has been shown in previous studies to be able to prevent the growth of cancer cells. This study aims to investigate the effects of metformin and gold nanoparticles in MCF7 breast cancer and A549 lung cell lines. The effects of metformin and gold nanoparticles on MCF7 breast cancer and A549 lung cells were determined on cells grown in 24 h cell culture. MCF-7 and A549 cells were incubated for 24 h with the treatment of escalating molar concentrations of ifosfamide. The MTT assay was used to determine the cytotoxicity of metformin toward MCF7 and A549 cell lines. The expression of Bax, BCL2, PI3K, Akt3, mTOR, Hsp60, Hsp70, and TNF-α was measured by RT-PCR. Metformin and gold nanoparticles inhibited the proliferation of MCF-7 and A549 cells in a dose and time-dependent manner with an IC50 value of 5 µM and 10 µg/mL. RT-PCR assays showed ifosfamide + metformin + gold nanoparticles significantly reduced the expression of BCL2, PI3K, Akt3, mTOR, Hsp60 and Hsp70 and increased the expression of TNF-α and Bax. The findings obtained in this study suggest that further studies should be conducted, and metformin and gold nanoparticles can be used in breast cancer and lung cancer treatments.

## Introductıon

The most frequent type of cancer-related death worldwide is breast and lung cancer [56, 78]. More than half of breast cancer patients will develop metastases to the bone, liver, lung, or brain [59]. The American Cancer Society has predicted that there will be around 127,070 deaths from lung cancer and about 238,340 new cases of lung cancer in the US in 2023 (https://www.cancer.org/). In addition, there will be 55,720 new instances of ductal carcinoma in situ (DCIS), 297,790 new cases of invasive breast cancer in women, and 43,700 new breast cancer-related deaths. Chemotherapeutic drugs that are effective in treating a wide range of malignant disorders include ifosfamide, carboplatin, cisplatin, etoposide and paclitaxel [13]. Ifosfamide, a cyclophosphamide analog, exhibits broad-spectrum action against a variety of neoplasms in various oncologic specialties, including haematological, breast and lung cancer [8, 57].

The first-line treatment for type 2 diabetes is the biguanide drug metformin, also known as 1,1-dimethylbiguanide hydrochloride. Many findings made in recent years have shown metformin's new function [44, 55]. Although metformin, a first-line medication for T2DM, is being employed for its anticancer properties, there is little information in the literature about how much metformin affects patients' overall survival when they have stage IV cancer [58]. Combined treatments of metformin and doxorubicin (DOX) are effective in the treatment of a variety of cancers, including breast cancer [61]. In studies, metformin significantly decreased the risk of bladder, oesophageal, and lung cancer [64].

Early tumor detection and diagnosis are the main foundations for using nanotechnology in treating cancer [60]. Due to their excellent electrical conductivity, stability, simplicity of modification, and biocompatibility, gold nanoparticles (AuNPs) have emerged as one of the most widely utilized materials in electrochemical biosensors, in medicinal and biological applications (K. X. [38, 72]. Gold nanoparticles have grown in significance in the realm of biomedical research and diagnostics due to their distinct physicochemical characteristics [29, 42]. There is hope and possibility for using gold nanoparticles in cancer treatment and diagnosis. However, it is crucial to consider unforeseen consequences for human health [60]. A study reports the first successful synthesis of highly stable gold nanoparticles using the bioactive compound naringenin in isolation, serving as a dual reducing and stabilizing agent [48]. It is important to note that AuNPs may influence cellular responses without affecting their viability, for example, inhibiting proliferation, altering calcium [25] and nitrogen oxide release [26], stimulating respiratory activity or the activity of mitochondrial enzymes [62]. AuNPs have the potential to be cytotoxic to specific cancer cell line types [65]. In vivo, AuNPs prevented VEGF-induced permeability and angiogenesis in mouse ovarian and ear tumor models [4, 46]. Gold nanoparticles that have internalized may modify intracellular signaling and obstruct the MAPK pathway, which would prevent metastasis by interfering with the epithelial-mesenchymal transition (EMT) [3].

A research was demonstrated that naturally occurring GA can be used as a nontoxic phytochemical construct in the production of readily administrable biocompatible AuNPs for diagnostic and therapeutic applications in nanomedicine [32]. AuNPs-based contrast agents may be useful in x-ray-based computed tomography [6]. Other reports have shown an unprecedented 82% reduction in tumor volume after a single-dose administration of GA-198AuNPs (408 μCi) [12]. The oncological implications of MGF-198AuNPs as a new therapeutic agent for treating prostate and various solid tumors are presented [30, 31]. Khoobchandani et al. [33] have achieved in clinically translating, from mice to humans, in using proprietary combinations of gold nanoparticles and phytochemicals to develop the Nano-Ayurvedic drug: Nano Swarna Bhasma (NSB), for treating human metastatic breast cancer patients [33]. The antitumor mechanism induced by YF-AuNPs on PC-3 and MDAMB-321 cell lines was attributed to apoptosis. Also, the results demonstrated that RAW 264.7 macrophages treated with YF-AuNPs resulted in elevated levels of antitumor cytokines (TNF-α and IL-12) and reduced levels of pro-tumor cytokines (IL-6 and IL-10) [68].

To our knowledge, in this exploration, the effects of metformin and gold nanoparticles on various genes in MCF7 and A549 cells were investigated for the first time in the literature. Thus, it was tried to reveal the anticarcinogenic effects of metformin and gold nanoparticles.

## Materıals and Methods

### Synthesis and Characterization of Citrate-capped Gold Nanoparticles

The synthesis of citrate-capped gold nanoparticles was performed using the procedure given in the literature [70]. In summary, a solution of 500 ml of 1 mM HAuCl_4_ in distilled water was taken into a one-liter glass flask and stirred until it boiled. A solution of 50 ml of 38.8 mM sodium citrate (Na_3_C_6_O_7_.2H_2_0) in distilled water was added to this solution quickly and stirring was continued for 10 min in boiling state, then it was mixed for 15 min without heating by warming from the heater. The solution, which turned from yellow to light red, was filtered at room temperature with small-pored filter paper and stored in the dark, ready to use.

Characterization of Au nanoparticles was observed using Ultraviolet–visible spectroscopy (UV–Vis) (Perkin Elmer Lambda 35 spectrophotometer device), which operated within the wavelength range of 200 to 900 nm. Functional groups were examined using Fourier transform infrared spectroscopy (FT-IR) with a Perkin Elmer Frontier FT-IR spectrophotometer, operating within a wavelength range of 400 to 4000 cm^−1^. For transmission electron microscopy (TEM) (Hitachi HT-7700 was employed), operating at 300 kV. Scanning electron microscopy (SEM) images were recorded on a FEI Inspect S50 SEM microscope operating at 25 kV.

### Cells Culture

MCF-7 (ATCC®HTB-22) breast cancer cell from a 69-year-old female patient and lung cancer cell with A549 (ATCC®CRM-CCL-155) epithelial cell type from a 58-year-old male patient were used in the study. MCF-7 cells in RPMI 1640 (Eco-Tech, Cat No: RPMI500) medium containing 10% FBS (Serana, Lot: 34010720FBS), 1% Penicillin/Streptomycin; it was grown at 5% CO_2_ and 37 °C ambient conditions. In the study, the effects of metformin and gold nanoparticles on MCF-7 and A549 cells were investigated by applying different concentrations. These changes were tested by comparing them with the control group that received no substance. For this purpose, considering the literature information researched, metformin was applied as 5 mM, 25 mM, 50 mM and 80 mM. The efficacy of gold nanoparticles was investigated by applying 5 µM, 25 µM, 50 µM and 100 µM concentrations. Ifosfamide is an alkylating antineoplastic agent widely used to treat different types of malignancies, including solid tumors and hematological malignancies [5, 74]. In this study, we applied Ifosfamide to cells at four different concentrations (1 μM, 5 μM, 25 μM, 50 μM, 75 μM).

### Measurement of Cell Cytotoxicity (MTT)

MTT (Measurement of cell cytotoxicity) test was performed to evaluate the effects of metformin and gold nanoparticles on viability by evaluating cell metabolic activity in MCF-7 and A549 cells. Cells reaching 70–80% density were seeded in 96-well plates at 1 × 10^3^ cells per well in four replicates for each concentration. After 24 h after sowing the cells, the medium was removed, and metformin and gold nanoparticles were applied at the determined concentrations. The MTT test was performed 24 h after the substances were administered. For this, the medium was removed and the mixture prepared at a ratio of 1:10 (Media: Cell Viability Detection Kit 8 (CVDK-8)) was added to the cells. Incubation was carried out at 5% CO_2_ and 37 °C for three hours and measurements were made at 450 nm. The results were calculated statistically using Microsoft Office Excel.

### RT-PCR Analysis

After MTT analysis, the effective dose for metformin was determined as 5 mM, Ifosfamide as 5 μM, and gold nanoparticle as 10 μM (Table 1). RT-PCR analyses were performed by applying these doses. Cells reaching 70–80% confluency were counted on the thoma slide and seeded at 1 × 10^6^ cells per well in 6-well plates. After 24 h, the substances were applied at the determined concentrations. At the end of 24 h and 48 h, the cells were removed with Trypsin–EDTA (Gibco, Ref:25,200–056), and RNA was isolated for RT-PCR.

**Table 1:** The dose and groups of treatment

Treatment Group	Dose
Control	-
Ifosfamide	5 µM
Metformin	5 mM
Gold Nanoparticles (AuNPs)	10 µM
Ifosfamide + Metformin	5 µM + 5 mM
Ifosfamide + AuNPs	5 µM + 10 µM
Metformin + AuNPs	5 mM + 10 µM
Ifosfamide + Metformin + AuNPs	5 µM + 5 mM + 10 µM

### RNA Isolation

Cells removed with trypsin were centrifuged at 5000 rpm for five minutes and the supernatant was removed. It was washed with 1 mL of phosphate-buffered saline (PBS) and centrifuged again. After this step, the RNA isolation kit (Ambion PureLink RNA Mini Kit Cat. Nos. 12183018A, 12,183,025) protocol was applied. RNA concentrations were measured at 260 nm. When the concentrations were around 100 and 260/280 values were around 2, the cDNA synthesis stage was started.

### cDNA Synthesis

cDNA synthesis was performed according to the Maxime RT PreMix Kit (Cat No. 25082) protocol. 5µL of RNA samples, with a final volume of 20µL, and 15µL of RNA-free water are added to the PCR tubes and placed in the PCR device. At the end of the PCR, the samples were stored at -20 °C until use.

### RT-PCR

The effects of the applied substances at the gene level was determined by real time PCR method. The cDNA synthesized was added to the mixture prepared with ddH2O, master mix, reverse primer of the gene to be studied and forward primer, with a total volume of 20µL. The tubes were spun and placed in the RT-PCR device. Gene analyses were performed at the end of the period. The effects of metformin and gold nanoparticles in controlled cell death apoptosis and PI3K, AKT3, mTOR, Bax, Bcl-2, Hsp60, Hsp70 and Tnf-a genes, which are genes involved in PI3K/Akt signaling pathway, were investigated by RT-PCR. The delta-delta Ct method (2^–∆∆Ct^ method) is a formula used to analyze the gene expression.

### Statistical Analysis

GraphPad Prism 8.01 was used to analyze all of the data. The normal distribution of the data was shown by the Shapiro–Wilk normality test. In this study, the statistical analysis of the MTT experiment, which was performed in four repetitions, was calculated using the Microsoft Office Excel program, and the concentration-dependent changes compared to the control group were examined. Taking these changes as a reference, the following tests were started. Afterwards, the RT-PCR experiment was performed and the analyses were calculated using Microsoft Office Excel. The Kruskal–Wallis test was used to analyze the distribution of gene expression results by groups. Statistics were considered significant for values with a p-value of 0.05.

## Results

### Characterization of Citrate-capped Gold Nanoparticles

**Fig. 1:**
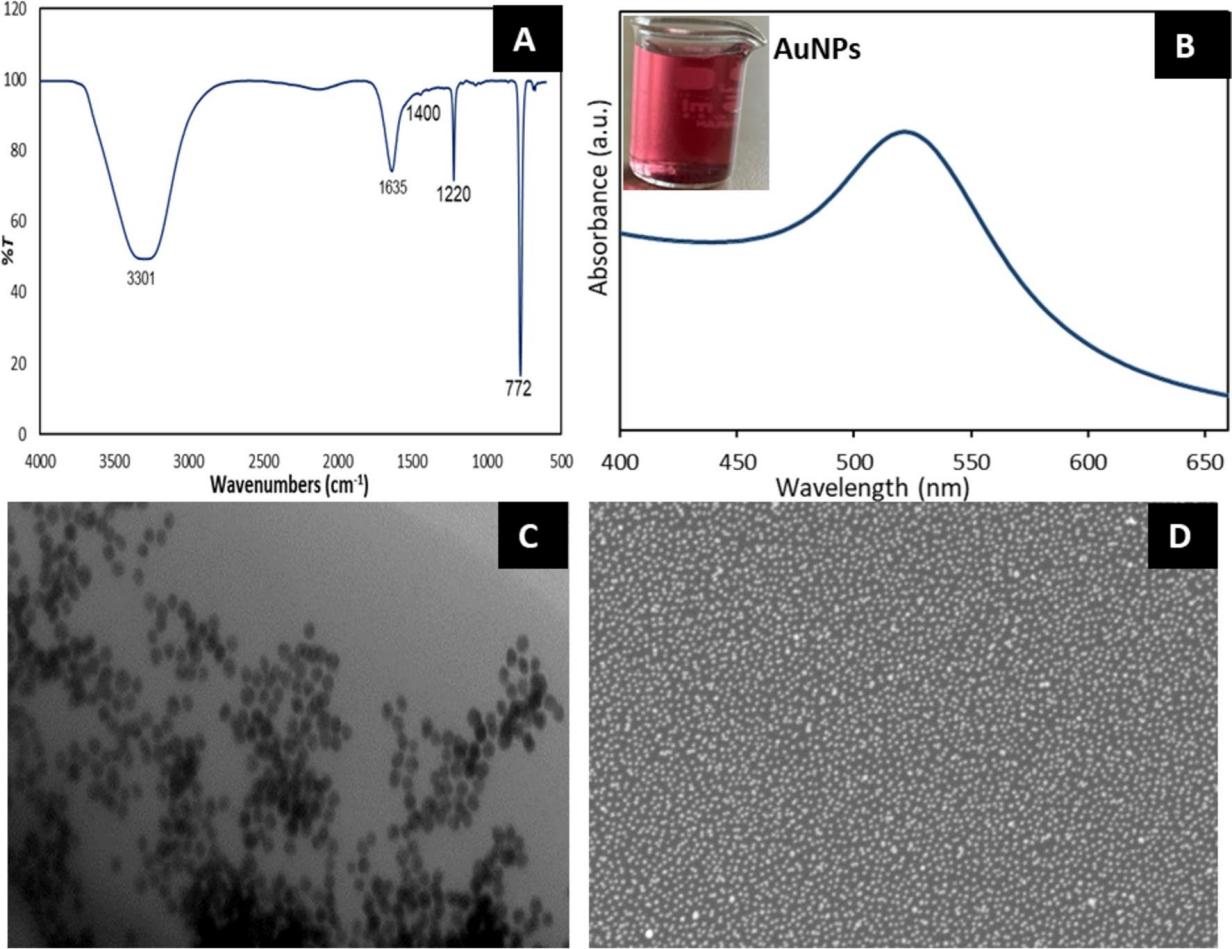
The FT-IR spectrum of citrate-capped gold nanoparticles **(A)**, UV spectrum of citrate-capped gold nanoparticles **(B)**, The transmission electron microscopy (TEM) image of citrate-capped gold nanoparticles **(C)**, Scanning electron microscopy (SEM) images of citrate-capped gold nanoparticles **(D) **

FT-IR, UV, TEM, and SEM spectroscopies were used to characterize citrate-capped gold nanoparticles. Citrate ions binding to gold nanoparticles was confirmed by FT-IR Analysis. The synthesized citrate-capped gold nanoparticles have a characteristic peaks corresponding to a citrate group and the presence of water molecules at 3301 cm ^−1^ (O–H), 1635 cm^−1^ (C = C, C = O), 1400 cm^−1^ (COO^−^) 1220 cm^−1^ (C–O), and 772 cm^−1^ (C–C) (Fig. 1.A). The O–H group can be attributed to the presence of water molecules and O–H stretching of citrate molecules in the sample, while C = C, C = O, C–O and C–C confirm the presence of citrate molecules along with the citrate-capped gold nanoparticles [69],Park and Shumaker-Parry, 2014; [71]. The presence of negatively charged citrate molecules for gold nanoparticles has critical functions. One of them is that it helps achieve chemical stability by lowering the surface energy of highly active nanoparticles. Another property is that they stabilize the nanoparticles, preventing their agglomeration and ensuring good dispersion of the nanoparticles, which is essential for their interaction with other molecules [83]. In UV analysis, the strong absorbance of the obtained citrate-capped gold nanoparticles solution at approximately 520 nm (ruby red color). The citrate-capped gold nanoparticles showed a typical peak at 520 nm under UV–visible spectroscopy, which further confirmed the synthesis of citrate-capped gold nanoparticles having sizes less than 20 nm [7] (Fig. 1.B). In addition, the TEM image of synthesized citrate-capped gold nanoparticles is shown in Fig. 1.C. The average size of citrate-capped gold nanoparticles was 14 nm and has a spherical morphology. Scanning electron microscopy (SEM) images of citrate-capped gold nanoparticles are shown in Fig. 1.D. SEM analysis, in addition to size the distribution of the synthesized citrate-capped gold nanoparticles appears to be quite well dispersed, while the average size of the citrate-coated gold nanoparticles is 10–20 nm.

### Anti-proliferative Effects of metformin and gold nanoparticles in MCF7 and A549 Cells

**Fig. 2:**
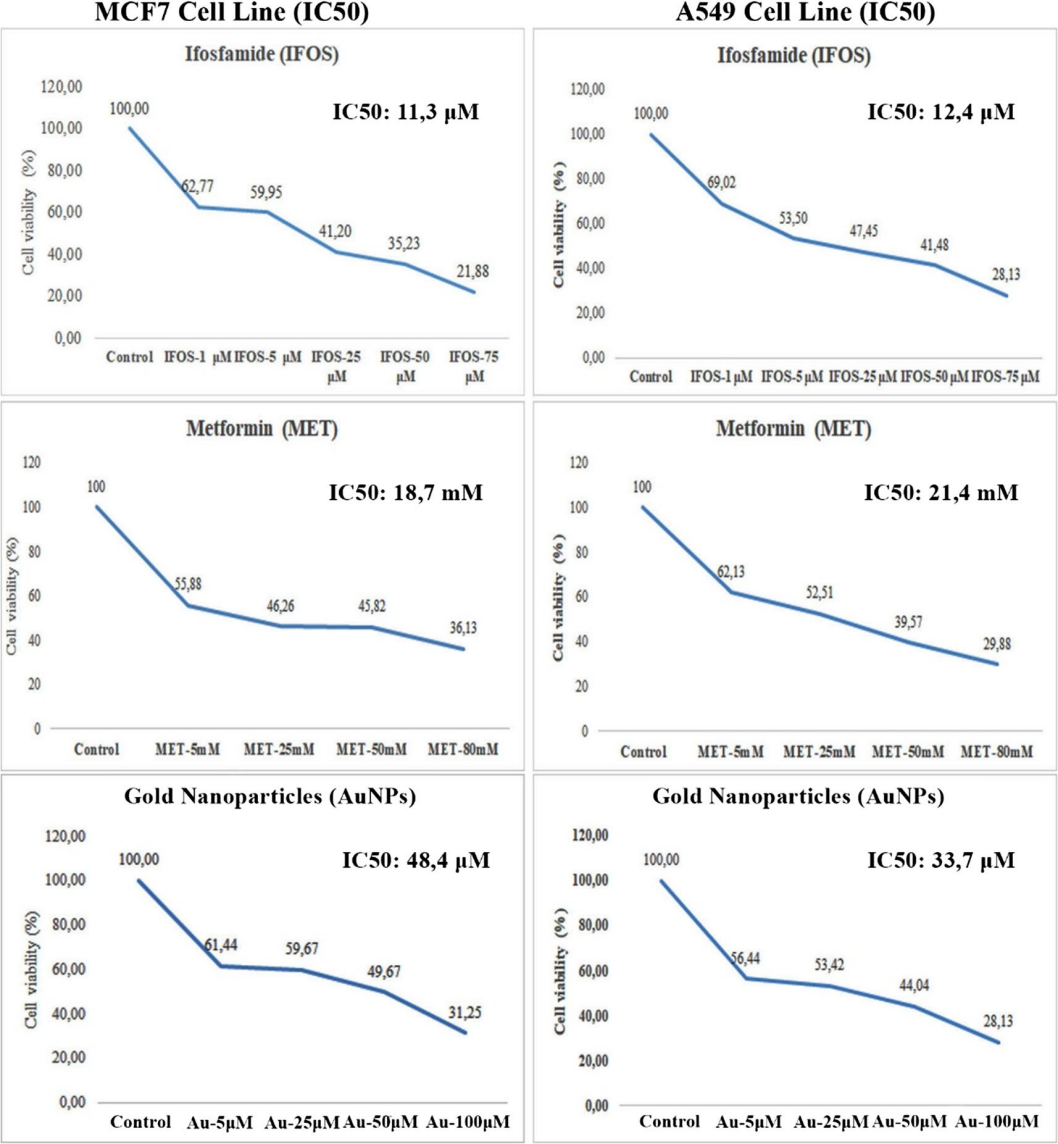
Cell viability (%) after treatment of metformin, ifosfamide and gold nanoparticles for 24 h on MCF7 and A549 cell lines

Different doses of ifosfamide (1 μM, 5 μM, 25 μM, 50 μM, 75 μM), metformin (5 mM, 25 mM, 50 mM and 80 mM) and gold nanoparticles (5 µM, 25 µM, 50 µM, 100 µM) were applied to MCF7 and A549 cells. The ability of metformin and gold nanoparticles to inhibit the proliferation of MCF7 and A549 cancer cell lines was determined by MTT assay for 24 h. As shown in Fig. 2, metformin and gold nanoparticles inhibited the cell viability of MCF7 and A549 cancer cells in a time- and dose-dependent manner.

The MCF-7 and A549 cell viability in control without any treatment was the highest. The treatments with ifosfamid, metformin and gold nanoparticles reduced the cell viability in the 5 mM metformin and 10 µM gold nanoparticles concentrations. The IC50 value for MCF-7 and A549 cells is presented in the Fig. 2.

Since it was observed that the 5 mM dose of metformin and 10 µM gold nanoparticles inhibited the proliferation of tumor cells, 5 mM metformin was preferred as the drug dose in the following parts of the experiments and expression studies were conducted.

### Determination of the Effects of Metformin and Gold Nanoparticles on Gene Expression Level in MCF7 and A549 Cells by RT-PCR

**Fig. 3:**
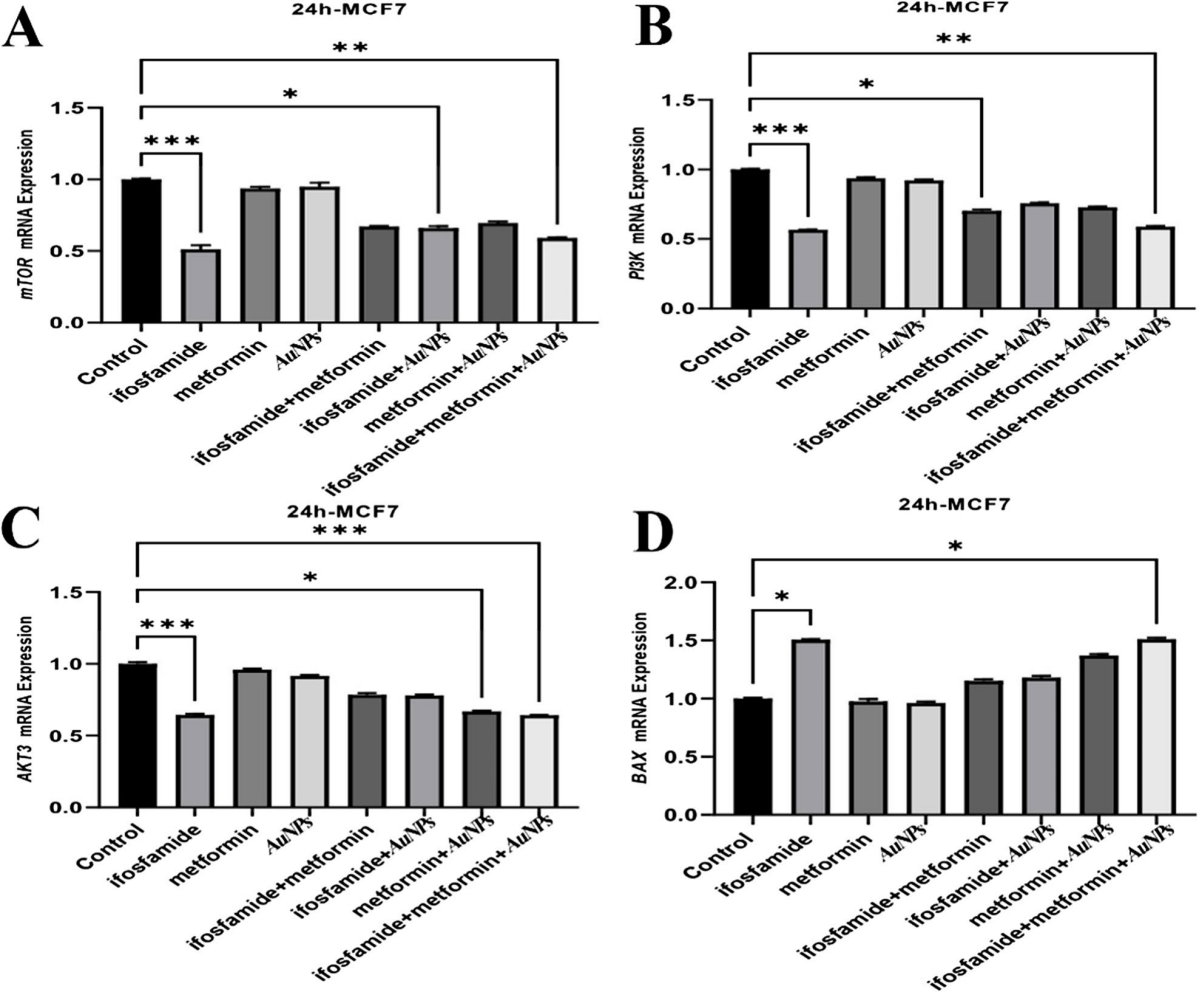
Gene Expressions of mTOR, PI3K, AKT3, and Bax in MCF-7 cells

**Fig. 4:**
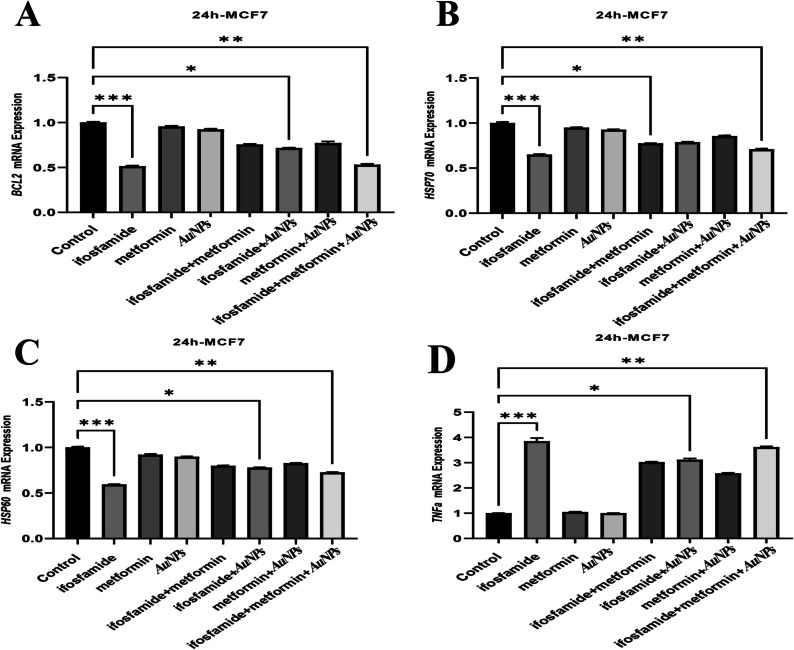
Gene Expressions of BCL2, HSP70, HSP60, and TNF-α in MCF-7 cells

The PI3K, AKT3, mTOR, BCL2, Hsp60 and Hsp70 mRNA expression levels were decreased by the ifosfamide + metformin + AuNPs treatment in MCF7 cell line. Also, the expressions of Bax and TNF-α increased by the ifosfamide + metformin + AuNPs treatment compared to the control (Fig. 3 and 4).

**Fig. 5:**
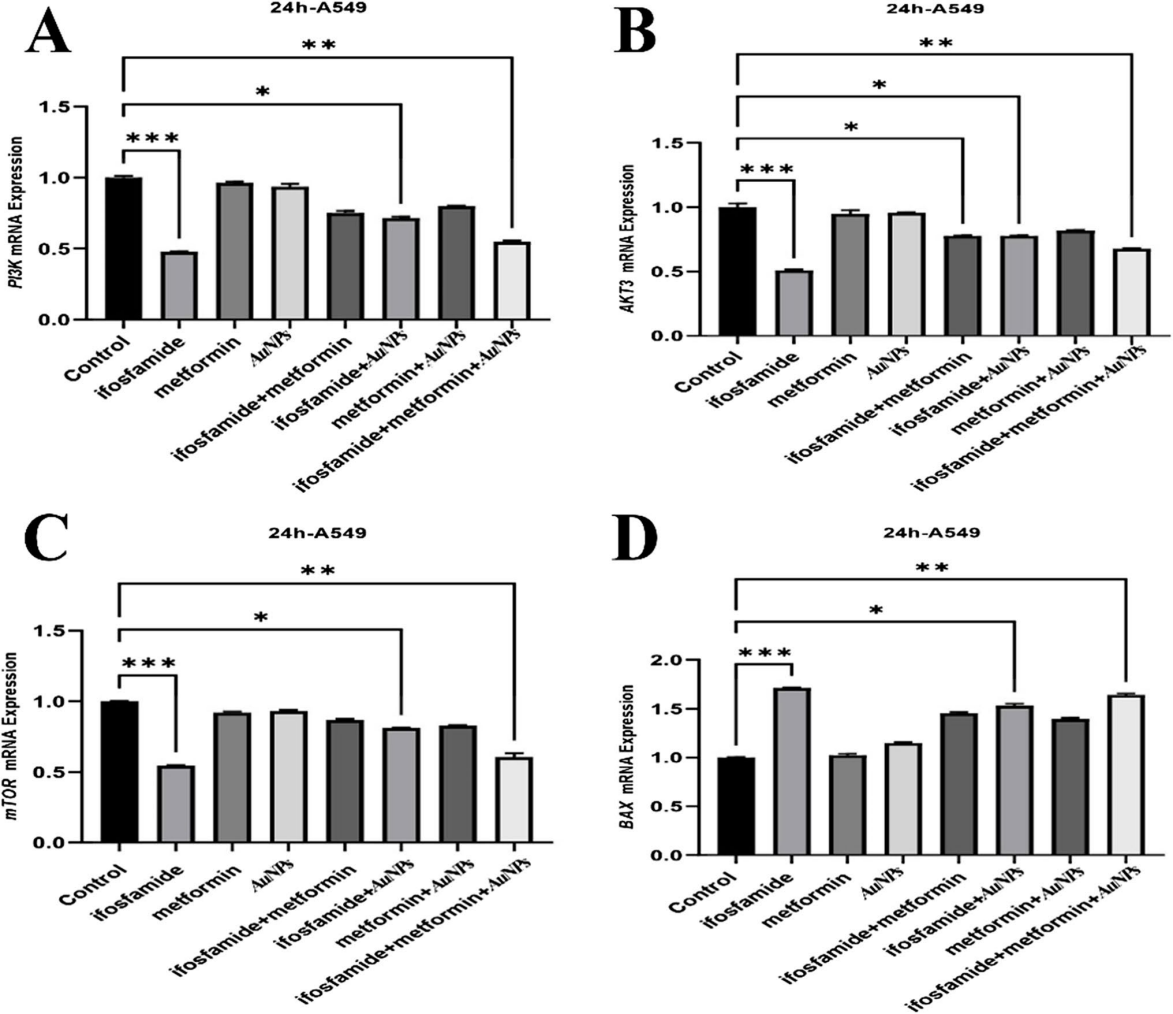
Gene expressions of PI3K, AKT3, mTOR and Bax in A549 cells

**Fig. 6:**
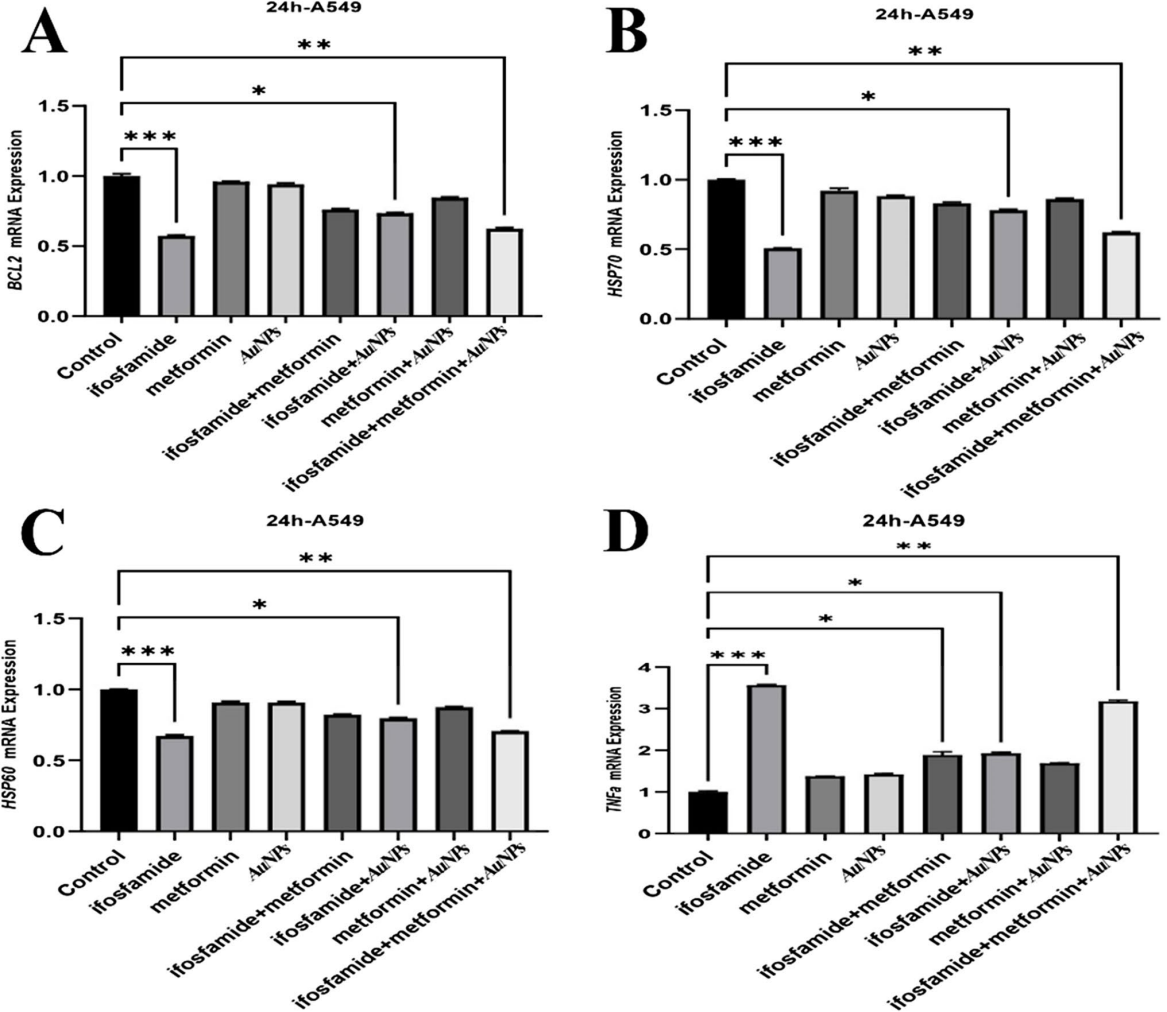
Gene expressions of BCL2, HSP70, HSP60, and TNF-α in A549 cells

The PI3K, AKT3, mTOR, BCL2, Hsp60 and Hsp70 mRNA expression levels were decreased by ifosmamide + AuNPs group and ifosfamide + metformin + AuNPs articles treatment in A549 cell line. Also, the expressions of Bax and TNF-α were increased by the ifosfamide + AuNPs group and ifosfamide + metformin + AuNPs treatment compared to the control (Fig. 5 and 6).

## Discussion

To our knowledge, this investigation is the first in vitro report of the effects of metformin and Au nanoparticles against human MCF7 and A549 cancer cells in the literature.

Tumors are related to decreased cell apoptosis and uncontrolled cell proliferation. Some chemotherapy drugs, including doxorubicin, cyclophosphamide, and paclitaxel, work like conventional cancer treatments by causing the tumor cells to die in an immune-mediated manner [36, 82].

Metformin has been used extensively in research, including in vivo cancer animal models and several cancer cell lines [2, 15, 84]. First receiving attention in 2005, metformin is an oral anti-diabetic medication that is more affordable than any other anticancer treatment now in use [17]. In case–control studies involving patients with breast cancers [27] and lung cancers [67], using metformin as an adjuvant to standard chemotherapy and radiotherapy has produced encouraging results. Metformin was reported to target cancer-initiating stem cells (R. [40]. Metformin may have an impact on tumorigenesis both directly and indirectly through the systemic lowering of insulin levels [49]. How to successfully eradicate cancer cells while leaving healthy cells unharmed is one of the main issues facing cancer research. We can create new medicines by analyzing the key molecular pathways underlying metformin's anticancer effects and the main types of death it mediates (J. [79].

In MCF-7 cells, metformin displayed an antiproliferative effect that depended on time and concentration. Compared to 2.5, 5 and 10 mM of metformin, this impact was stronger at 20 mM [53, 79] showed that metformin could effectively inhibit the proliferation of some breast cancer cells in dose- and time-dependent manner (J. [79]. Metformin therapy inhibited cell growth dose-dependently in MCF-7 and MCF-7/713 cell lines. Also, MCF-7 cells were 57% and MCF-7/713 cells were 50% less likely to proliferate at a dose of 50 mM compared to untreated controls [1]. Metformin was used at a concentration that does not affect the growth of non-transformed cells (0.1 or 0.3 mM). Metformin was previously utilized at significantly greater concentrations (usually 10–30 mM) in investigations on cancer cell lines [1]. In a study, the scientists looked at metformin's antiproliferative effects in MCF7 cancer cells and its mechanism of action in MCF-7 cancer cells exposed to 10 mM of the drug for 24, 48, and 72 h. It was detected that metformin showed a time- and concentration-dependent antiproliferative impact in MCF-7 cells [53]. In this study, it was shown that 5 µM metformin and 10 µM Au nanoparticles were effective in suppressing the proliferation of MCF7 and A549 in groups.

Many tumor types have been discovered to be inhibited by metformin. Its impact on non-small cell lung cancer (NSCLC) is still unknown. Metformin might prevent A549 and H1299 cells from proliferating, according to a study [41]. A considerable reduction in cell proliferation and significant activation of apoptosis were seen in A549, RERF-LC-A1, IA-5, and WA-hT cells after exposure to metformin (1–20 mM) [66]. Metformin is predicted to exert anticancer effects by inhibiting the insulin and mTOR pathways [51]. According to previous studies, the relationship between metformin use and lung cancer survival is unclear [87]. It has been reported that the use of metformin alone or in combination with existing chemotherapy may be a good approach to managing lung cancer effectively [23]. One study examined the relationship between autophagy and apoptosis in the A549 lung cancer cell line using metformin (6 mM) and gedunin (12 µM), an inhibitor of Hsp90 [24]. According to the results of this study, metformin and gedunin have cytotoxic effects against A549 lung cancer cells [24]. It has been reported that the malignant properties of A549 and H3122 cells can be inhibited by metformin in vitro [52]. Metformin has been shown to have an IC50 of 13.5 mM and 21.8 mM against A549 and H3122 cells, respectively. In this study, the findings suggest that 5 µM metformin and 10 µM Au nanoparticles were effective in suppressing the proliferation of A549 cells in groups.

In MCF-7 cells, metformin has been reported to decrease IRβ, Akt and ERK1/2 activation and phosphorylation of p70S6K and Bcl-2 protein expression, increase p-AMPK, FOXO3a, p27, Bax and cleaved caspase-3 [53]. According to findings in molecular and cellular studies, metformin has been reported to increase p53 and Bax levels significantly and decrease STAT3 and Bcl-2 [43]. Apoptosis was markedly accelerated by 10 mM metformin [73]. In this study, MCF 7 cells caused a significant decrease in Bax mRNA expression in the groups treated with ifosfamide + metformin + AuNPs. In addition, the BCL2 gene caused a significant decrease in Bcl-2 mRNA expression in the groups treated with ifosfamide + AuNPs and ifosfamide + metformin + AuNPs to MCF 7 cells. Metformin has been shown to reduce A549 or H1651 cell growth and invasive capacity in vitro. It was also determined that Bax significantly reduced Ser184 phosphorylation, Myc's Ser62 phosphorylation, and Akt's Ser473 phosphorylation [88]. In the current study, there was an increase in mRNA expression of the Bax gene in metformin A549 cells in ifosfamide + AuNPs and ifosfamide + metformin + AuNPs groups, while BCL2 mRNA expression decreased.

One of the main mechanisms of action of metformin is the activation of adenosine monophosphate-activated protein kinase (AMPK). AMPK is associated with the PI3K/PTEN/AKT pathway and MAPK/ERK [34]. It has been suggested that simultaneously targeting AMPK using metformin and the PI3K/AKT/mTOR pathway by an mTOR inhibitor may become a new therapeutic approach [34]. One study shows that metformin inhibits EGF-induced EMT in MCF-7 cells, possibly associated with the PI3K/Akt/NF-κB signaling pathway [45]. Metformin, flavone and co-treatment have been shown to have no effect on AKT1 expression in MDA-MB-231 and MCF-7 cells [85].

An increase in AKT3 expression was detected at low frequencies in breast carcinomas, gliomas and hepatocellular carcinomas [35, 54]. mTOR is known to be abnormally activated in cancers as it plays an important role in regulating metabolism [39]. In this study, a statistically significant decrease was found in PI3K mRNA expression in the groups treated with ifosfamide + metformin and ifosfamide + metformin + AuNPs. It was determined that the mRNA expression of the AKT3 gene was lower in the metformin + AuNPs and ifosfamide + metformin + AuNPs groups compared to the control group. Also, mRNA expression of the mTOR gene was low in ifosfamide + AuNPs and ifosfamide + metformin + AuNPs groups.

Combining metformin and celecoxib therapy may cause apoptosis in A549 cells by blocking the ERK and PI3K/AKT signaling pathways [11]. Celecoxib and metformin both prevented PI3K/AKT signaling. AKT phosphorylation was scarcely eliminated by combination therapy [11]. In this study, it was determined that PI3K mRNA expression in A549 cells decreased significantly in the ifosfamide + AuNPs and ifisfamide + metformin + AuNPs groups compared to the control. AKT3 mRNA expression was significantly decreased in ifosfamide + metformin, ifosfamide + AuNPs and ifosfamide + metformin + AuNPs groups. There was a significant decrease in mTOR gene expression in the ifosfamide + AuNPs and ifosfamide + metformin + AuNPs groups.

Cancer cells produce heat shock proteins (Hsps) in response to exposure to thermal and other proteotoxic stresses [80]. In addition to thermal stress, HSPs also protect them from exposure to oxidative stress, chemical, physical and other stresses [63]. HSP27, 60 and 70 play a crucial role in apoptotic processes at the mitochondrial level [9, 10, 19] and represent important targets for the development of drugs.

HSPs have been reported to be abnormally expressed in different types of cancer, including breast, colorectal, and lung (J. H. [37]. Poor clinical outcomes have been linked to particularly high levels of Hsp27, Hsp70, and Hsp90. Intracellular and cell surface HSP70s are recognized as potential targets for the treatment of breast cancer [28]. Most mammalian cells have large amounts of Hsp60, essential for protein folding and chaperoning (Frydman & Hartl, 1996). Compared with healthy breast tissues, HSP60 mRNA level has been shown to be significantly increased in primary breast cancer tissues [16]. Elevations in circulating HSP70 have been reported in association with malignant transformation, including breast cancer [21, 81]. In one study, it was emphasized that HSP70 could be used in addition to other diagnostic tests for breast cancer and could be useful in demonstrating the risk of breast cancer [22]. We could not find any study showing the effect of metformin on HSP60 and HSP70 genes in breast cancer. However, the effect of metformin on HSP in other cancer cells has been investigated. Metformin has also been shown to reduce the expression of Bcl-2 and HSP27, HSP60 and HSP70 [50]. Metformin has been reported to increase NK cell cytotoxicity by regulating the mRNA and protein expression of MICA and HSP70 on the surface of human cervical cancer cells via the PI3K/Akt pathway [76]. Metformin has been shown to reduce HSP70 [73]. A decrease in the mRNA expression of the HSP60 gene was observed in the groups treated with ifosfamide + AuNPs and ifosfamide + metformin + AuNPs to MCF7 cells. On the other hand, it was shown that there was a decrease in HSP70 mRNA expression in ifosfamide + metformin and ifosfamide + metformin + AuNPs groups.

It has also been noted that HSP60 expression is associated with the onset of lung cancer [77]. In this study, it was determined that both the HSP60 gene and HSP70 gene mRNA expression in A549 cells were statistically significantly decreased in ifosfamide + AuNPs and ifisfamide + metformin + AuNPs groups than the control.

It has been shown that proinflammatory cytokines IL-6 and TNF-α are significantly increased in enhancing the inflammatory cascade in patients with metastatic breast cancer [20]. In MDA-MB231 and MDA-MB453 breast cancer cells treated with metformin, the expression of IL-12 and TNF-a cytokines was increased [14]. It has been reported that there is a significant increase in the secretion of TNF-a, IL-2 and IFN-y cytokines in NSCLC with metformin. [75]. TNF-α mRNA expression was statistically significantly higher in the groups treated with ifosmaid + AuNPs and ifosfamide + metformin + AuNPs to MCF7 cells. On the other hand, in A549 cells, there was a significant increase in TNF-α expression in the groups treated with ifosfamide + metformin, ifosfamide + AuNPs and ifosfamide + metformin + AuNPs.

## Conclusion

In conclusion, the findings of this study showed that metformin and gold nanoparticles inhibited MCF7 and A549 cell proliferation. 5 µM ifosfamide, 5mM dose of metformin and 10µM gold nanoparticles cause cell death in cells by inducing cell cytotoxicity, inflammation, and apoptosis. Our findings suggest that using metformin and gold nanoparticles as a promising chemotherapeutic agent for treating human breast and lung cancer requires further investigation.

## Data Availability

Data are contained within this article or supplementary material.
