# Correction to: Anticarcinogenic Effects of Gold Nanoparticles and Metformin Against MCF-7 and A549 Cells

**DOI:** 10.1007/s12011-024-04155-y

**Published:** 2024-04-04

**Authors:** Ali Yeşildağ, Halime Topal Kızıloğlu, Ebubekir Dirican, Elif Erbaş, Volkan Gelen, Adem Kara

**Affiliations:** 1 Department of Bioengineering, Faculty of Engineering and Architecture, Kafkas University, Kars, Turkey; 2 Department of Molecular Biology and Genetic, Faculty of Science, Erzurum Technical University, Erzurum, Turkey; 3 Department of Medical Biology, Faculty of Medicine, Bilecik Şeyh Edabali University, Bilecik, Turkey; 4 Department of Histology and Embryology Faculty of Veterinary Medicine, Atatürk University, Erzurum, Turkey; 5 Department of Physiology, Faculty of Veterinary Medicine, Kafkas University, Kars, Turkey

**Correction to: Biological Trace Element Research**

10.1007/s12011-024-04090-y

The published version of this article unfortunately contained mistakes.

Figures 2 to 6 in the original version of this article has been replaced.

**Corrected Fig. 2.** 
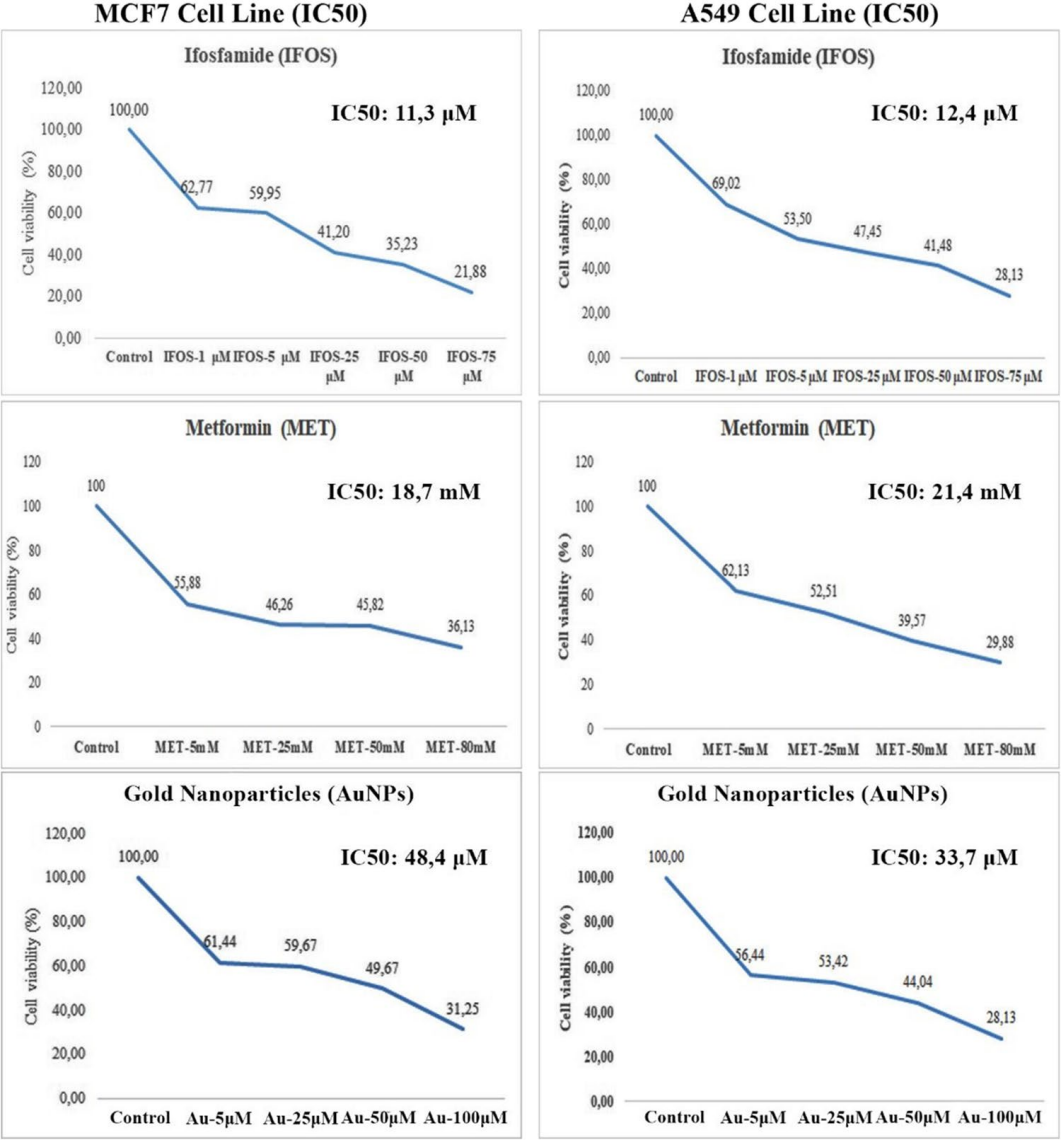


**Corrected Fig. 3.** 
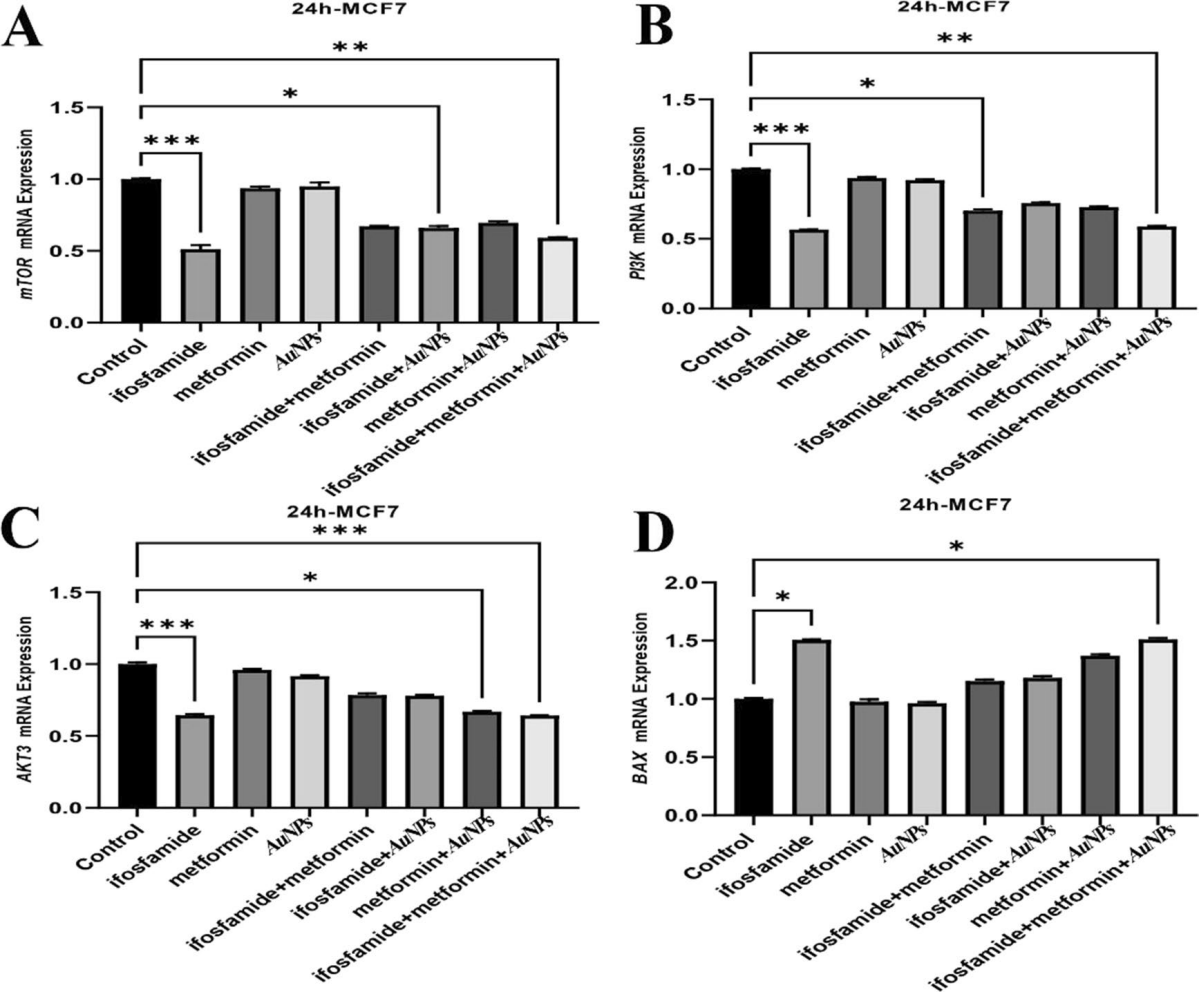


**Corrected Fig. 4** 
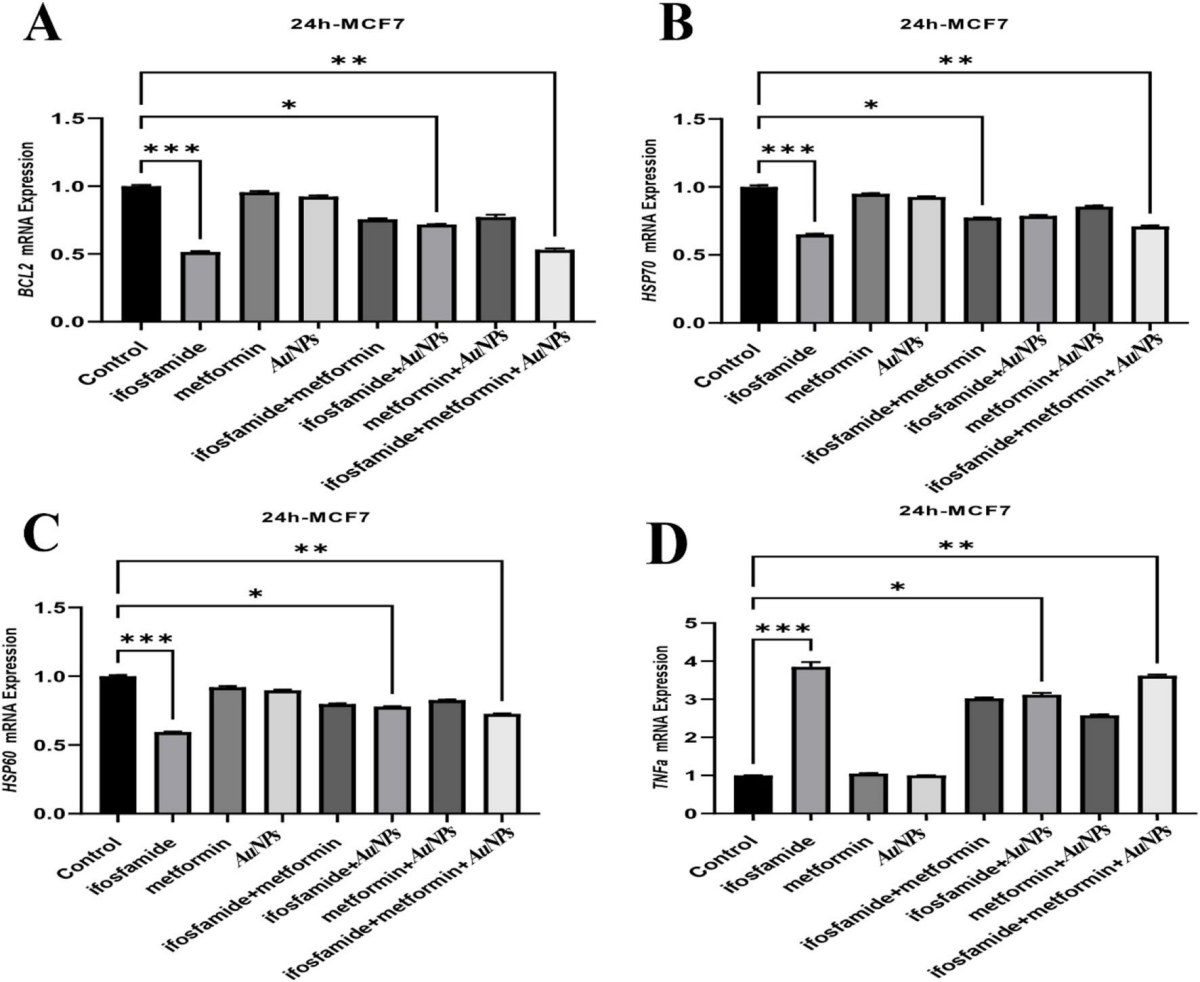


**Corrected Fig. 5** 
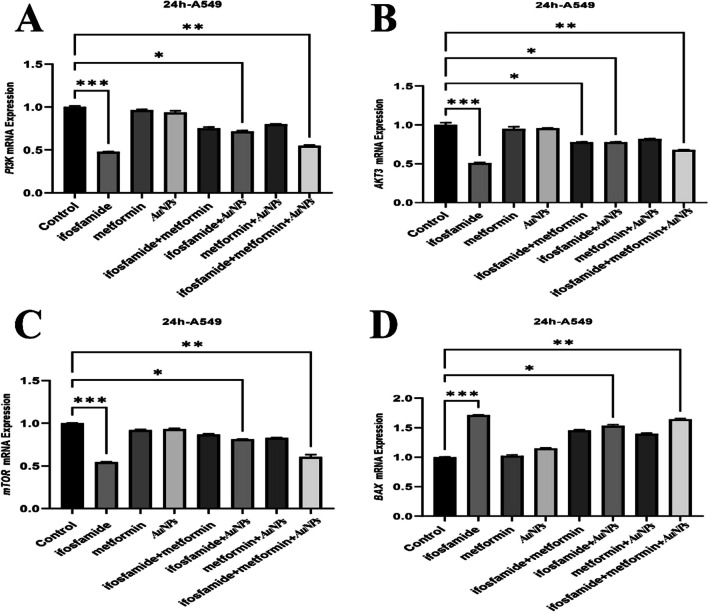


**Corrected Fig. 6** 
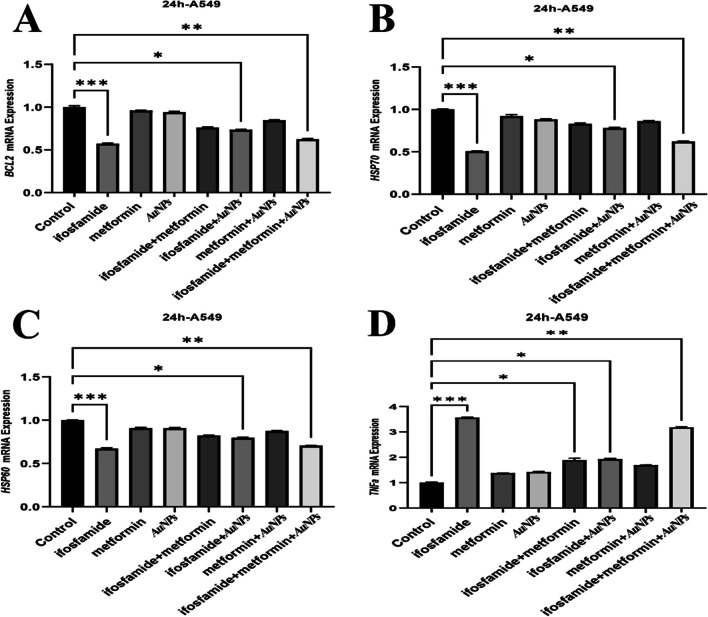


The original article has been corrected.

